# Mapping Chemical Elements and Iron Oxidation States in the Substantia Nigra of 6-Hydroxydopamine Lesioned Rats Using Correlative Immunohistochemistry With Proton and Synchrotron Micro-Analysis

**DOI:** 10.3389/fnins.2019.01014

**Published:** 2019-09-26

**Authors:** Asuncion Carmona, Stéphane Roudeau, Laura Perrin, Carole Carcenac, Delphine Vantelon, Marc Savasta, Richard Ortega

**Affiliations:** ^1^UMR 5797, Chemical Imaging and Speciation, CENBG, University of Bordeaux, Gradignan, France; ^2^UMR 5797, CNRS, IN2P3, CENBG, Gradignan, France; ^3^INSERM U1216, Physiopathologie de la Motivation, Grenoble, France; ^4^Grenoble Institute of Neuroscience, Université Grenoble Alpes, Grenoble, France; ^5^SOLEIL Synchrotron, Saint Aubin, France; ^6^Centre Hospitalier Universitaire de Grenoble, Grenoble, France

**Keywords:** brain, imaging, Parkinson, metal, iron, manganese, synchrotron, PIXE

## Abstract

Brain metal homeostasis is altered in neurodegenerative diseases and the concentration, the localization and/or the chemical speciation of the elements can be modified compared to healthy individuals. These changes are often specific to the brain region affected by the neurodegenerative process. For example, iron concentration is increased in the substantia nigra (SN) of Parkinson’s disease patients and iron redox reactions might be involved in the pathogenesis. The identification of the molecular basis behind metal dyshomeostasis in specific brain regions is the subject of intensive research and chemical element imaging methods are particularly useful to address this issue. Among the imaging modalities available, Synchrotron X-ray fluorescence (SXRF) and particle induced X-ray emission (PIXE) using focused micro-beams can inform about the quantitative distribution of metals in specific brain regions. Micro-X-ray absorption near edge spectroscopy (XANES) can in addition identify the chemical species of the elements, in particular their oxidation state. However, in order to bring accurate information about metal changes in specific brain areas, these chemical imaging methods must be correlated to brain tissue histology. We present a methodology to perform chemical element quantitative mapping and speciation on well-identified brain regions using correlative immunohistochemistry. We applied this methodology to the study of an animal model of Parkinson’s disease, the 6-hydroxydopamine (6-OHDA) lesioned rat. Tyrosine hydroxylase immunohistochemical staining enabled to identify the SN pars compacta (SNpc) and pars reticulata (SNpr) as well as the ventral tegmental area (VTA). Using PIXE we found that iron content was higher respectively in the SNpr > SNpc > VTA, but was not statistically significantly modified by 6-OHDA treatment. In addition, micro-SXRF revealed the higher manganese content in the SNpc compared to the SNpr. Using micro-XANES we identified Fe oxidation states in the SNpr and SNpc showing a spectral similarity comparable to ferritin for all brain regions and exposure conditions. This study illustrates the capability to correlate immunohistochemistry and chemical element imaging at the brain region level and this protocol can now be widely applied to other studies of metal dyshomeostasis in neurology.

## Introduction

Metallic elements such as manganese (Mn), iron (Fe), and copper (Cu) are essential for human since they act as cofactors of many enzymes and participate in cell respiration and metabolism. However, biological essential metals can also be involved in redox reactions leading to the formation of reactive oxygen species (ROS) that promotes cell death. In the nervous system metal dyshomeostasis resulting in production of ROS, protein aggregation or more generally in cell injury may contribute to the etiology of neurodegenerative diseases. It is now well documented that metal dyshomeostasis is involved in the main neurodegenerative disorders, Alzheimer’s disease, Parkinson’s disease (PD), Huntington’s disease, amyotrophic lateral sclerosis and prion diseases (Barnham and Bush, 2014; Collingwood and Davidson, 2014; Ward et al., 2014; Toni et al., 2017). Modifications in metal content and/or their redox species are expected to occur locally in the pathological brains, in the most vulnerable brain regions affected by the pathology. The study of these local modifications requires the use of analytical techniques with microscopic spatial resolution for their observation (Barnham and Bush, 2014; Davies et al., 2014; Collingwood and Adams, 2017; Hackett et al., 2019).

Parkinson’s disease is characterized by the progressive loss of dopaminergic neurons in the substantia nigra pars compacta (SNpc) in the ventral midbrain. The etiology of PD is poorly understood and is multifactorial, with inputs from a variety of genetic, environmental and endogen factors, among them Fe dyshomeostasis. In PD, there is a specific increase of Fe concentration in the SNpc in comparison to age-matched controls that could be involved in the selective loss of dopaminergic neurons in the SNpc (for review: Sian-Hülsmann et al., 2011; Ward et al., 2014; Liddell and White, 2018). Increased nigral Fe has been proposed as a biomarker for early diagnostic of PD using magnetic resonance imaging (MRI) for Fe quantification in patients (Guan et al., 2017). Some studies have not observed such Fe increase in the SN of PD brains (reviewed in: Friedman and Galazka-Friedman, 2012), reflecting the complexity of this assessment. Another interesting feature about Fe region-specific distribution in the SN is that Fe content is higher in the SNpr of healthy individuals, the region of the SN that is not affected by neuronal loss in PD (Drayer et al., 1986; Morris and Edwardson, 1994; Morawski et al., 2005). Animal models such as the 6-hydroxydopamine (6-OHDA) lesioned rat have been developed to study PD. 6-OHDA induces a progressive anterograde degeneration of the nigrostriatal pathway, the main anatomical feature in PD. This animal model might be helpful to reproduce some features of Fe dyshomeostasis in PD, in particular the increase of Fe in the SNpc (He et al., 1996; Wang et al., 2004; Hare et al., 2009, 2010; Dexter et al., 2011). For all the above mentioned reasons it is now needed to investigate the distribution of Fe in specific brain regions using micro-analytical methods able to distinguish for instance between the SNpc and the SNpr thus requiring adequate spatial resolution, especially in animal models where the size of the SN is smaller than in human brains.

X-ray microanalysis using proton or synchrotron radiation microbeams can be employed to image Fe and other elements content with great sensitivity and quantitative accuracy within local microscopic regions (Ortega et al., 2009; Collingwood and Davidson, 2014; Pushie et al., 2014; Collingwood and Adams, 2017). In addition, micro-XANES can be used to identify Fe(II) and Fe(III) from differences in their absorption edge energies and pre-edge features (Ortega et al., 2012; Collingwood and Davidson, 2014; Collingwood and Adams, 2017; Porcaro et al., 2018). The determination of chemical element distributions in brain regions requires the use of non-denaturating sample preparation methods usually based on cryogenic protocols of tissue fixation. The classical histology protocols based on chemical fixation and staining of biological tissues are known to disturb the native distribution and speciation of the elements (Chwiej et al., 2005; Ralle and Lutsenko, 2009; Hackett et al., 2011; James et al., 2011; Robison et al., 2012; Davies et al., 2014; Roudeau et al., 2014; Perrin et al., 2015; Jin et al., 2017).

The aim of this study was first to develop a methodology allowing the exact location of the chemical elements in the brain regions of interest (SNpc, SNpr, and VTA) while preserving the native element distribution and speciation. This aim was achieved by comparing element imaging to tyrosine hydroxylase (TH) immuno-histochemical staining from adjacent tissue cryo-sections. Then our second objective was to perform quantitative PIXE analysis and XANES Fe oxidation state speciation in the SN of 6-OHDA lesioned rats at the microscopic level to clearly distinguish between the SNpc SNpr, and VTA regions. We measured the quantitative distribution of Fe and of some other elements (P, S, Cl, K, Ca, Cu, and Zn) in each brain area and for the two brain hemispheres, the ipsilateral (IpsiL) 6-OHDA lesioned, and the contralateral (ContraL) non-lesioned side. We also compared the results with those obtained on control animals (sham) injected with a NaCl solution without 6-OHDA.

## Materials and Methods

### Animal Experiments

Animal experiments were carried out according to the guidelines for the care and use of laboratory animals approved by the European Community Council Directive of 24 November 1986 (86/609/EEC), and the ethics committee of the Grenoble Institute of Neuroscience. One month old Wistar rats were anesthetized with a mixture of xylazine (15 mg/kg, i.p.) and ketamine (100 mg/kg, i.p.) 30 min before intracraneal 6-OHDA injection (6-Hydroxydopamine hydrobromide, H116, Sigma). Rats were secured in a Kopf stereotaxic apparatus (Phymep, Paris, France) and 1 μL of 6-OHDA (injured animals) or 1 μl of sterile 0.9% NaCl (sham conditions), were unilaterally injected in the SNpc at a flow rate of 0.5 μL/min. Two weeks later rats were anesthetized by inhalation of isoflurane and the dorsal skull was exposed. Brains were perfused to avoid vascularization. In order to preserve structure and chemical composition after extraction, brains were cryofixed by rapid plunging into a cooled cryogenic liquid (isopentane cooled with dry ice at −40°C), and then stored at −80°C. The correct location of the injection cannula was checked by collecting the SN using the atlas of Paxinos and Watson (2007). Both sides of the brain were studied, the ipsilateral (IpsiL, injured side) and the contralateral (ContraL, control side). Samples were obtained from 15 6-OHDA lesioned animals and 12 sham animals. 6-OHDA administration resulted in a 35–85% (mean 60%) range decrease in TH expression as measured by TH immuno-histochemistry.

### Sample Preparation: Overview

**FIGURE 1:**
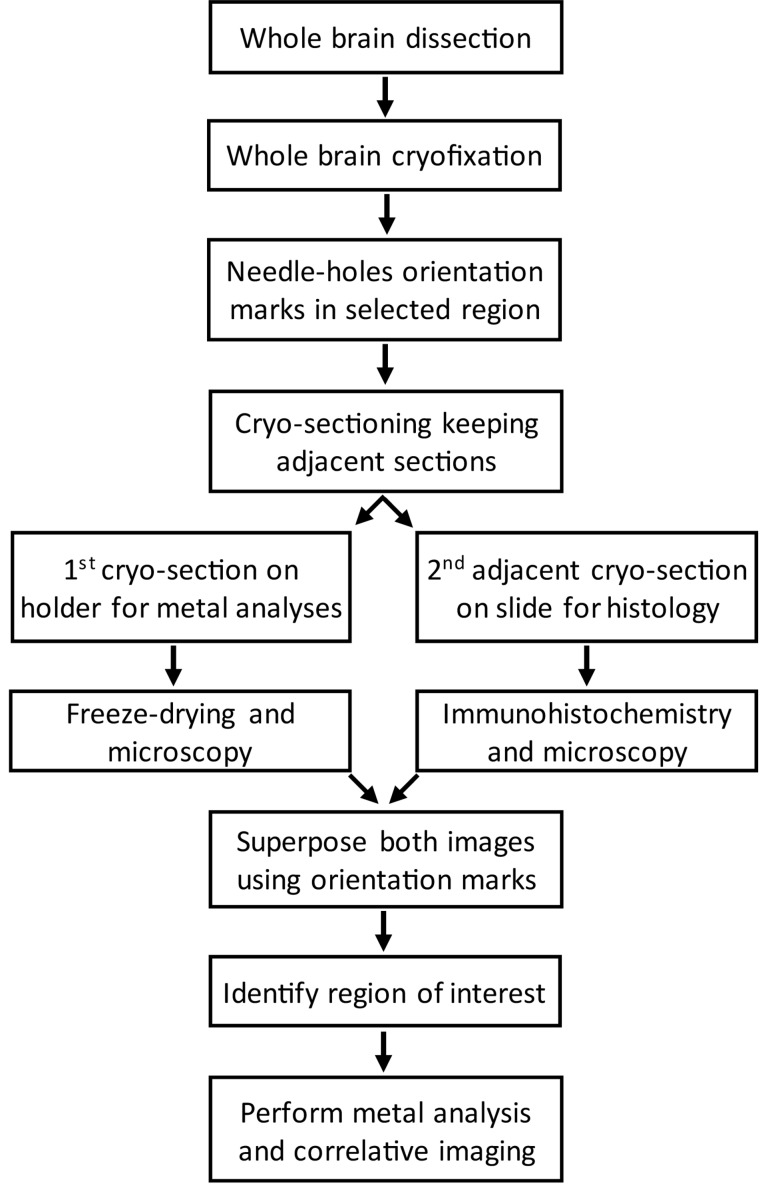
Workflow of the procedure to correlate brain regions from cryo-sections stained for TH by immunohistochemistry with unstained cryo-sections prepared for metal imaging.

Our aim was to determine the elemental content in specific regions of the rat brain: SNpc, SNpr and VTA. The location of these specific regions in the brain can be performed by immunohistochemical staining of the dopamine synthesis enzyme TH, as explained in the next section. However, immunohistochemistry preparations based on chemical fixation and staining are not suited for chemical imaging since they are known to alter elemental distribution and speciation (Chwiej et al., 2005; Ralle and Lutsenko, 2009; Hackett et al., 2011; James et al., 2011; Robison et al., 2012; Davies et al., 2014; Roudeau et al., 2014; Perrin et al., 2015; Jin et al., 2017). To make both purposes compatible we used cryofixed animal brains and performed histological staining and chemical analysis on consecutive, adjacent, brain tissue cryo-sections. By superposition of both cryo-sections we could determine the areas of interest, in this case the localization of SNpr, SNpc, and VTA, in the unstained sections used for X-ray microanalysis. In order to identify precisely the brain regions of interest several important steps need to be considered before sectioning. The workflow of the procedure is illustrated in Figure 1.

**FIGURE 2:**
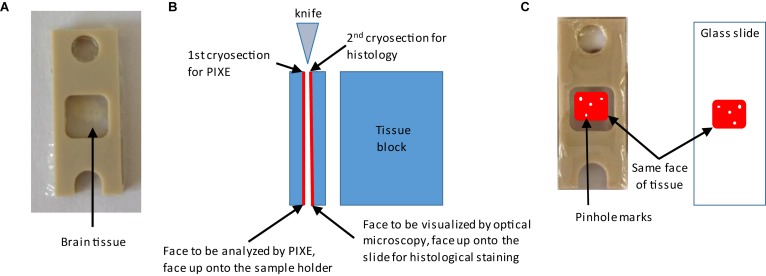
Procedure for the preparation of adjacent brain cryo-sections. Needle holes orientation marks are made around the region of interest on the tissue block before sectioning. **(A)** A first brain cryo-section is deposited and maintained between two polycarbonate films stretched over a PEEK sample holder. This first cryo-section will be used for metal analysis. **(B)** Explanation of how to keep the tissue orientation for correlative imaging. The second cryo-section is used for immunohistochemistry. This second cryo-section is deposited on a glass microscopy slide with the face adjacent to the first one on the top. **(C)** Illustration of how to identify regions of interest on both sections thanks to the needle holes orientation marks.

Two adjacent cryo-sections are cut: one for the elemental analysis (PIXE, XANES, or SXRF) and the other one for histological staining. Cryo-sections for histological staining are deposited onto usual microscope slides. For PIXE, XANES, or SXRF analyses, glass slides cannot be used since they contain element impurities and thus an appropriate sample holder must be chosen. Several materials are available such as silicon nitride membranes or thin polymers films of ultrapure quality (Perrin et al., 2015). In this study we used a polycarbonate film of 2 μm thickness stretched on a plastic frame holder made of PEEK (Figure 2A). Both brain cryo-sections must have a common face to perform correlative imaging. This is the face that is cut by the knife when performing the first cryo-section as highlighted in red in Figure 2B. This face should be deposited top up on the sample holders, the PEEK/polycarbonate sample holder and the microscope slide, as illustrated in Figure 2C. To superimpose precisely the images of the two adjacent cryo-sections, we used orientation marks consisting in some small pinholes made with a needle in the tissue before sectioning. By superposition of the holes, the region of interest can be identified on both cryo-sections, as schematically shown in Figure 2C.

### Preparation of Brain Cryo-Sections

The frozen tissue block was transferred into the cryo-microtome and mounted with tissue freezing medium for cryo-sectioning at −25°C. For PIXE, XANES, and SXRF analyses semi-thin 50 μm cryo-sections of brain tissues were prepared. If the tissue section is too thin, the metal content might not be detectable or leading to too long acquisition times to perform the experiments. If the tissue section is too thick then some metal contribution could come from deeper brain regions below the region of interest. The polycarbonate film on the PEEK sample holder was placed into the cryostat at −25°C to cool down before to deposit the cryo-section. Once the tissue and the sample holder reach the right temperature, the cryo-section is deposited onto the polycarbonate/PEEK sample holder, keeping the face to be analyzed up. To avoid tissue deformation, the cryo-section is covered with a second PEEK/polycarbonate sample holder. By this mean, the brain cryo-section is maintained between the two polycarbonate films in a stable position during handling and analysis. A second 20 μm thickness cryo-section immediately adjacent to the previous one is placed onto a microscope glass slide for histological staining. Similarly, to the previous step, the slide is placed into the cryostat to reach the correct temperature, and the brain cryo-section is deposited keeping the common face up (Figure 2).

Depending on the analytical method that will be further used, the initial 50 μm cryo-sections prepared for elemental analysis are either freeze dried for PIXE analysis, in order to be placed under vacuum, or stored in cryotubes placed in liquid nitrogen until XANES and SXRF analyses which are performed with a sample cryostage on frozen hydrated samples. For PIXE analysis, cryo-sections were freeze dried with a Christ alpha 2–4 LD plus at −85°C under vacuum (2.5 × 10^–3^ mbar) during 24 h and slowly rewarmed to room temperature under vacuum.

### TH Immuno-Histochemical Staining

Brain tissue sections from the whole brain, IpsiL and ContraL sides, were mounted on silane-coated microscope slides. Tissue sections were postfixed in 4% paraformaldehyde, thoroughly twice washed with Tris buffered-saline (TBS, 0.1 M, pH 7.4) and incubated for 1 h in 0.3% Triton X-100 in TBS (TBST) and 3% normal goat serum (NGS, Sigma-Aldrich, St. Quentin Fallavier, France) at room temperature. They were then incubated with primary antisera diluted in TBST supplemented with 1% NGS overnight, at 4°C. The antiserum was diluted 1:500 for TH staining (mouse monoclonal antibody; Chemicon, Temecula, CA, United States). Antibody binding was detected with avidin-biotin-peroxidase conjugate (Vectastain ABC Elite, Vector Laboratories, Burlingame, CA, United States), with 3,3’-diaminobenzidine as the chromagen. The detection reaction was allowed to proceed for 1–3 min. Sections were dehydrated in a series of graded ethanol solutions, cleared in xylene, mounted in DPX (DBH Laboratories Supplies, Poole, United Kingdom) and covered with a coverslip for microscopy (Favier et al., 2013).

### Identification of Brain Regions

**FIGURE 3:**
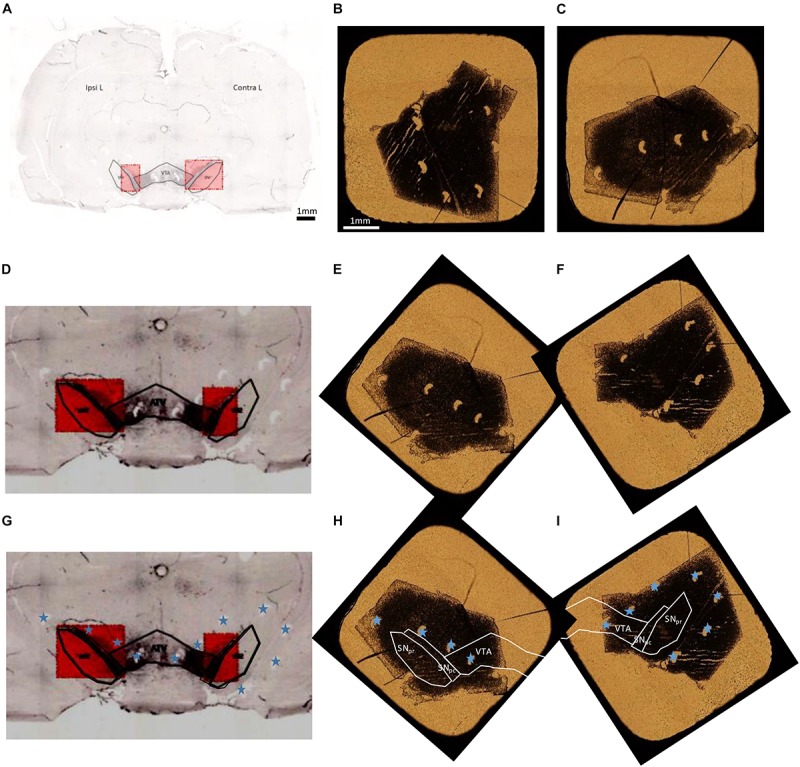
Overall procedure for superposition of TH immuno-histochemical sections and the adjacent freeze dried unstained section for PIXE analysis. **(A)** Entire brain section stained with TH to localize the dopaminergic pathway, SNpc, SNpr, and VTA. **(B,C)** Optical transmission microscopy images of freeze dried sections for PIXE analyses (**B:** IpsiL brain side and **C:** ContraL brain side). **(D,G)** Zoom of the stained region, to distinguish the pinholes done around the SN (indicated with blue stars). These images have a horizontal flip with respect to the initial image in order to obtain the same orientation for the pinholes than on freeze dried samples. **(E**,**F)** PIXE section images, applying a rotation, to display the same orientation of pinholes than on the immuno-histochemical sections. **(H,I)** Correctly oriented images using the pinholes (marked with a blue star) to locate precisely the SNpc, SNpr, and VTA regions on the sections for PIXE analysis. Scale bars = 1 mm.

Tyrosine hydroxylase immuno-labeled and unstained adjacent freeze dried sections are observed by optical transmission microscopy and the images of the whole sections are reconstructed. Superposition of the histological image reconstruction and of the optical microscopy image of the freeze dried sample are obtained by superposition of the pinhole orientation markers made before sectioning. The areas of interest in the immuno-histochemical section are used to determine the localization of these areas in the freeze dried sections. The overall procedure for the superposition of histological images and optical microscopy images of freeze dried samples is illustrated in Figure 3.

### Micro-PIXE Analyses

Micro-PIXE analyses were performed at CENBG using a 3.5 MV Singletron particle accelerator (HVEE, Netherlands) at AIFIRA facility (Applications Interdisciplinaires des Faisceaux d’Ions en Région Aquitaine). Micro-PIXE and micro-RBS (Rutherford Backscattering Spectrometry) analyses were carried out simultaneously, using the high resolution microbeam line, with a proton beam of 3.0 MeV, focused at 2 μm spatial resolution and resulting in a 0.8 nA beam current. Emitted X-rays were collected using two lithium drifted silicon X-ray detectors (Sirius, United Kingdom), placed at 135° on both sides of the beam axis direction. Backscattered particles were collected using a passivated implanted planar silicon detector (Canberra, France) placed at 135° below the incident beam direction.

The microbeam setup for 3 MeV protons allows to scan square surfaces of maximum 750 μm side. Therefore, for each tissue section, we performed several analyses in order to cover all the surface of the regions of interest, SNpc, SNpr, and VTA, as previously described for the analysis of large tissue sections (Carmona et al., 2017). Thanks to the superposition of the images of TH staining and of the adjacent freeze dried section, we could exactly delimit the regions of interest to be analyzed by PIXE (Figure 3). Then PIXE and RBS local spectra corresponding exactly to the brain regions of interest (SNpr, SNpc, VTA) were extracted post-analysis from each of the individual 750 μm× 750 μm scan. Element concentration expressed in μg.g^–1^ were determined for each of the selected regions. Finally, the elemental content in the three regions of interest (SNpc, SNpr and VTA) was calculated as the mean value of the extracted regions of all the individual scans performed on each region. The number of analyzed animals was 15 for the 6-OHDA group and 12 for the Sham group.

Quantitative data treatment of PIXE spectra was performed using the GUPIXWIN software (Campbell et al., 2010). This software allows determining the elemental areal mass (μg ⋅ cm^–2^). Charge normalization of PIXE data was obtained by fitting RBS spectra with SINMRA software (Mayer, 1997) following a methodology described in earlier publications (Carmona et al., 2008, 2017; Perrin et al., 2015). The areal mass of the tissues (g ⋅ cm^–2^) was also determined by RBS, this parameter is necessary to normalize the PIXE areal mass and express the element content as μg of element per g of dry mass (μg ⋅ g^–1^). Calibration of the X-ray detectors for quantitative analysis was performed using Micromatter^TM^ XRF calibration standards.

### Micro XANES and SXRF Analyses

Micro-XANES and micro-SXRF experiments were conducted at SOLEIL, LUCIA beamline (Flank et al., 2006; Vantelon et al., 2016). The beam was focused to 4.0 μm × 2.5 μm by means of mirrors installed in a Kickpatrick-Baez. Micro-XANES analyses were performed at Fe K-edge absorption energy, where the provided flux was 6.4 × 10^9^ ph ⋅ s^–1^. The spectra were acquired in fluorescence mode by scanning the near edge region (7050–7250 eV) at a rate of 10 s per energy step with a 0.2 eV energy step around the edge (7100–7160 eV), 2 eV below the edge (7050–7100 eV), and 1 eV above the edge (7160–7250 eV). Experiments were conducted on frozen hydrated tissue sections. Samples were maintained in a dried nitrogen atmosphere before the experiment and cooled by thermal contact with the cryo-stage (−196°C) during the measurements. The samples were prepared in the same way than for PIXE analyses but without freeze drying, and were kept refrigerated at all time in liquid nitrogen. Iron oxidation state in the SNpc and SNpr for 6-OHDA injured and sham animals were measured at least in triplicate. XANES spectra were calibrated in energy against the absorption edge position of Fe(0) metallic foil, whose first inflection point is 7112 eV. Three different reference compounds were analyzed: FeO as Fe(II) standard, Fe_2_O_3_ and ferritin as Fe(III) standards. Micro-SXRF imaging was performed after micro-XANES analyses. The energy was fixed at 7200 eV, the pixel resolution was 4 μm and the time of analyses par pixel was set to 1 ms.

### Statistical Analyses

Statistical analyses were conducted using open access R software (R Core Team, 2013), R Commander (Fox and Bouchet-Valat, 2018), and RStudio (RStudio Team, 2016). We compared each brain region (SNpc, SNpr, and VTA) and brain side (ContraL and IpsiL) for the two injection conditions (sham and 6-OHDA injured). The normality postulate was not always fulfilled and we considered the regions and brain sides as not paired. Multiples comparison of elemental content were performed using Kruskal Wallis test. Wilcoxon comparison as *post hoc* test was used to determine, for each element, which zone show significant differences, no method to adjust *p*-value was used (Feise, 2002).

## Results

### Element Content in Brain Regions Measured by Micro-PIXE

**FIGURE 4:**
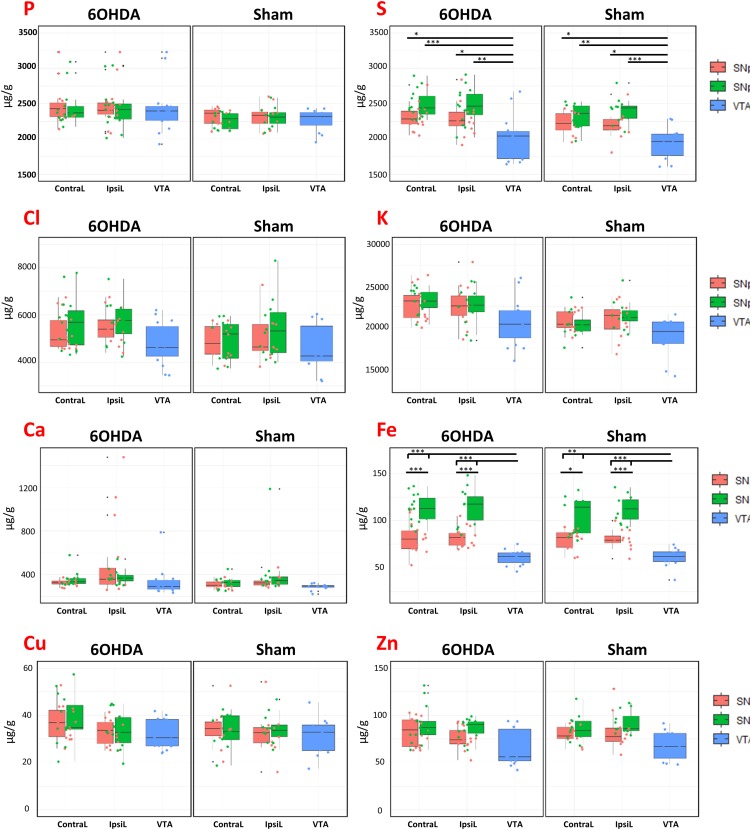
Boxplots of the element (P, S, Cl, K, Ca, Fe, Cu, Zn) concentrations (expressed in μg/g dry mass) for animals injected with 6-OHDA and Sham in the different brain regions (SNpc, SNpr, VTA) for both brain sides (IpsiL and ContraL). Boxplots represent the median and the first and third quantiles, the whisker represents the upper and lower values excluding the outliers. ^∗^0.005 < *p* < 0.001, ^∗∗^*p* < 0.001, and ^∗∗∗^*p* < 0.0001.

Particle induced X-ray emission technique, in combination with RBS, enables the quantification of the elemental content in biological tissues (Carmona et al., 2008, 2017; Perrin et al., 2015). We could determine P, S, Cl, K, Ca, Fe, Cu, and Zn in freeze dried brain tissues sections. We analyzed 15 animals injured with 6-OHDA and 12 control animals (sham, injected with a NaCl saline solution). The injection was unilateral, but both sides of the brain, IpsiL and ContraL, were analyzed. Three dopaminergic zones were distinguished in each side, SNpc, SNpr, and VTA. The quantitative results are presented in Figure 4 showing boxplots with the elemental concentrations, expressed in μg.g^–1^, for each brain region, for injured and control animals (6-OHDA and Sham, respectively), and in each side of the brain (ContraL and IpsiL).

When comparing the sides and regions of interest between lesioned animals and controls, Fe concentrations are unchanged (Figure 4). This result indicates that the injection of 6-OHDA did not significantly modified the Fe content in the SNpc, SNpr, and VTA of the lesioned animals despite a in a 35–85% (mean 60%) range decrease in TH expression. On the other hand, Fe content was found statistically significantly higher in the SNpr than in the SNpc for each brain side and for all conditions (Figure 4). Hence, we found that Fe content is well compartmentalized in the three dopaminergic regions, the lowest values were found in the VTA and the highest in SNpr. Statistically significant differences were also observed for sulfur, with a lower content in the VTA than in SNpc or SNpr in both 6-OHDA and Sham cases. Except for iron and sulfur no other important significant differences were noticed.

### Iron Oxidation State Measured by Micro-XANES and XRF Imaging of Fe and Mn

**FIGURE 5:**
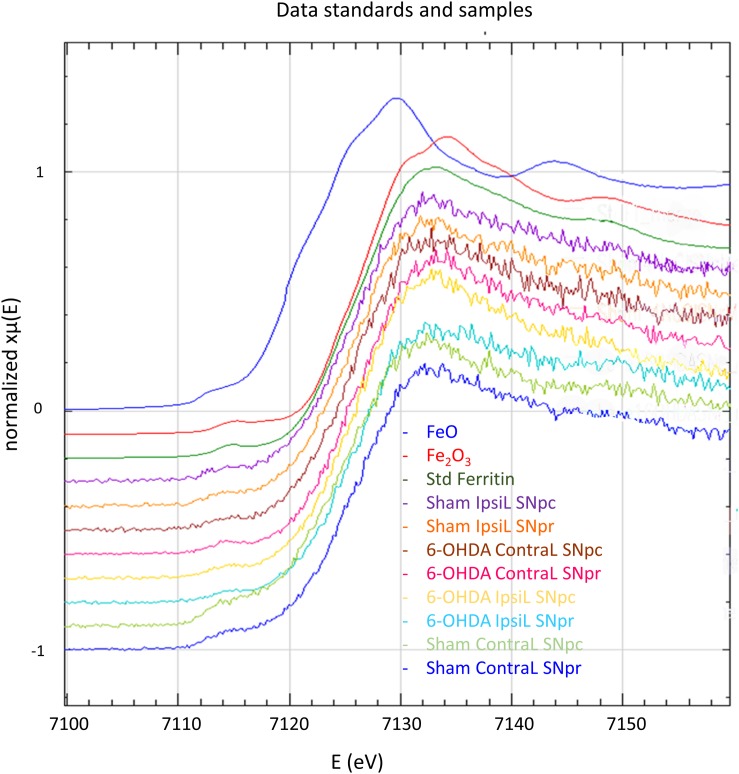
X-ray absorption near edge spectroscopy spectra at Fe absorption K-edge for FeO, Fe_2_O_3_, ferritin standards and in the SNpc and SNpr for the ContraL and IpsiL brain sides of 6-OHDA injured and sham animals. All the measured spectra on brain samples show the same shape as ferritin and Fe(III) standards.

Iron can be present in biological tissues in different oxidation states, mainly Fe(II) and Fe(III), which determine the reactivity and potential toxicity of this element. Iron oxidation state was measured in the SNpc and the SNpr and for both brain sides (IpsiL and ContraL) and injection conditions (6-OHDA and sham). Micro-XANES data indicated that Fe is present in form of Fe(III), in all cases, regardless of the injection side or the brain region (Figure 5 and Table 1). Micro-XANES spectra for all brain regions show that Fe exhibits very similar shapes and values of the first derivative inflection point as for the ferritin standard, suggesting that iron could be complexed principally to ferritin (Figure 5 and Table 1).

**TABLE 1:** Values of the XANES inflection point of the first derivative for each brain region analyzed, the inflection occurs at the same value as Fe(III) and ferritin standards and for all brain regions and injection conditions.

**Sample**	**EO (eV)**
Standard Fe(II)	7119.8 ± 0.5
Standard Fe(III)	7123.6 ± 0.5
Ferritin	7123.9 ± 0.5
Sham Ipsilateral SNpc	7123.3 ± 0.5
Sham Ipsilateral SNpr	7123.4 ± 0.5
Sham Contralateral SNpc	7124.2 ± 0.5
Sham Contralateral SNpr	7123.3 ± 0.5
60HDA Ipsilateral SNpc	7123.9 ± 0.5
60HDA Ipsilateral SNpr	7124.3 ± 0.5
60HDA Contralateral SNpc	7124.0 ± 0.5
60HDA Contralateral SNpr	7123.8 ± 0.5

**FIGURE 6:**
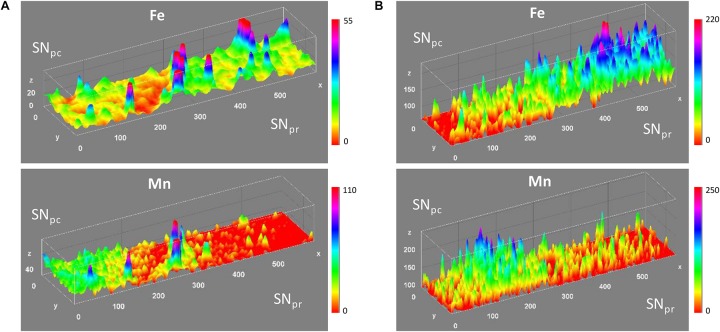
Representative micro-SXRF distribution maps of Fe and Mn in the SNpc, appearing at the left side of the images, and SNpr, right side of the images. **(A)** Distribution maps of Fe and Mn along the SNpc and SNpr in the ContraL side of sham rat brain. **(B)** Distribution maps of Fe and Mn in the ContraL side of a 6-OHDA injured rat. Similar distributions were found in other cases with no effect of 6-OHDA. *x* and *y* axis in μm; *z* axis in number of x-ray fluorescence counts.

Following micro-XANES analyses, micro-SXRF element mapping were performed on the same samples. The objective was to map Fe and Mn distribution at the junction of SNpc and SNpr. Mn was below the limit of detection for micro-PIXE analysis but could be detected by SXRF which is a more sensitive analytical method (Ortega et al., 2009). Similarly, to micro-PIXE results, micro-SXRF analyses confirmed the higher content of Fe in the SNpr vs. the SNpc (Figure 6). Contrary to Fe distribution, Mn intensity is higher in the SNpc than in the SNpr. This preferential Mn accumulation in the SNpc appears in sham and 6-OHDA injured animals and in both brain sides, IpsiL and ContraL.

## Discussion

Micro-analytical and spectroscopic imaging methods are required to quantify and probe oxidation states of metals in local areas of the brain. This need is particularly well illustrated in the case of the SN that is separated in two regions, SNpc and SNpr, with distinct Fe content. Iron histochemistry revealed that non-haem Fe staining was higher in the SNpr than in the SNpc in the human brain (Drayer et al., 1986; Morris and Edwardson, 1994). This result was further confirmed for total Fe using quantitative micro-PIXE measurements (Morawski et al., 2005). This Fe compartmentalization in the SN was also observed in mice using immuno-assisted laser ablation-inductively coupled plasma-mass spectrometry (Hare et al., 2014), or using a combination of LA-ICPMS and SXRF (Davies et al., 2015). Our results on Wistar rats are in agreement with these studies showing that that Fe content is higher in the SNpr than in SNpc and higher in the SNpc than in VTA. These results reproduce well the data obtained in human (Drayer et al., 1986; Morris and Edwardson, 1994; Morawski et al., 2005) and in mice (Davies et al., 2015), including the comparison with the adjacent VTA region (Hare et al., 2014). The higher Fe content in the SNpr vs. SNpc could be due to the presence of oligodendrocytes which are cells known to contain more Fe than neurons (Snyder and Connor, 2009).

Anomalous Fe handling has been proposed to be involved in the selective loss of dopaminergic neurons from the SNpc (Sian-Hülsmann et al., 2011; Ward et al., 2014). Using Fe histochemical assays, an increase of Fe has been reported in the SNpc from PD patients (Sofic et al., 1988; Riederer et al., 1989; Morris and Edwardson, 1994; Faucheux et al., 2003) which might contribute to ROS production and neuronal degeneration. However, the quantitative distribution and speciation of Fe in the SN relied mostly on semi-quantitative Fe histochemical methods, and these methods are not sensitive to all chemical species of the metal. Electron X-ray microanalysis confirmed Fe dyshomeostasis with the observation of increased Fe in the neurons from the SN of PD patients (Oakley et al., 2007), as well as SXRF showing the global increase of Fe in the SN of PD patients (Popescu et al., 2009), and in neuromelanin neurons (Davies et al., 2014). The 6-OHDA rat model has been suggested as an animal model to study Fe dyshomeostasis in PD. For instance, this animal model has been successfully used to study the effect of Fe chelation on the protection of dopaminergic neurons from the SNpc showing a neuroprotective effect of clinically available Fe chelators (Dexter et al., 2011). Initial observations using histochemical Fe staining have shown that the degeneration of nigrostriatal dopaminergic neurons following 6-OHDA lesions is associated with increased Fe accumulation in the SN of about 35% in the IpsiL SN vs. the ContraL SN (Oestreicher et al., 1994). This result was confirmed by a quantitative spectrometric approach based on micro-PIXE analysis showing a 26% increase of Fe in the SN from the 6-OHDA IpsiL lesioned-side of the animals (Watt et al., 1995). More recently, the use of quantitative LA-ICP-MS (laser ablation-inductively coupled plasma-mass spectrometry) also showed a 20% increase of Fe in the SN from the IpsL lesioned-side (Hare et al., 2010).

In our study, however, we did not observe any variation of Fe content following 6-OHDA treatment, in any of the studied brain regions (SNpc, SNpr, and VTA) (Figure 4). Compared to other studies, we used a relatively mild 6-OHDA treatment which resulted in a TH decreased expression ranging from 35 to 85% (mean 60%). These conditions were selected in order to study animals modeling mid-stages of PD progression. However, Fe increase in the SN might only be observed following extensive 6-OHDA lesions of the nigrostriatal dopamine pathway, leading to a complete or almost complete depletion of the dopaminergic neurons (Oestreicher et al., 1994; Olmedo-Díaz et al., 2017). In these conditions of complete dopaminergic depletion a 20–30% increase of total Fe is generally observed. In our conditions, we were expecting to observe slighter modifications proportional to the level of TH decrease. Our data indicate that the quantitative modification of Fe content was too low to be observed following mild injections of 6-OHDA. Our data also indicate that the 6-OHDA treated rat model of PD has some limitations to study Fe dyshomeostasis. Fe accumulation occurs only for very advanced stages of dopamine denervation suggesting that Fe increase might not be involved in the neurodegenerative process induced by 6-OHDA. The 6-OHDA rat model has, however, proven to be very useful to study advanced stages of dopamine neuron degeneration, reproducing some important features observed in PD such as the increase of Fe in the SNpc, to study neuroprotective effects of Fe chelators (Dexter et al., 2011). Other investigations have shown that the Fe increase is associated to a decrease in ceruloplasmin expression in the SN of 6-OHDA lesioned-rat (Wang et al., 2015). Ceruloplasmin is a ferroxidase which converts Fe(II) into Fe(III) and cooperates with ferroportin1 to export Fe from cells. Other studies also suggested that the increase of Fe in the SN could be provoked by the dysfunction of the blood brain barrier in the 6-OHDA rat model (Oestreicher et al., 1994; Virel et al., 2014; Olmedo-Díaz et al., 2017).

Since the total Fe content was unchanged following mild injections of 6-OHDA, we investigated if the 6-OHDA treatment could modify the ratio between Fe(II)/Fe(III) in the SN and be used as an animal model of pro-oxidative conditions related to PD. Some reports using Fe(II) histochemical assays support the Fe oxidation hypothesis in PD, showing the local increase of redox-active Fe(II) in neuromelanin neurons (Faucheux et al., 2003) and Lewy bodies from the SNpc of PD brains (Castellani et al., 2000). Mixed valences of Fe(II)/Fe(III) have been observed in a single neuron from a PD patient using micro-XANES (Yoshida et al., 2001). There is also one report on animal models, mixed valences of Fe(II)/Fe(III) were observed using micro-XANES on the MPTP primate model of PD showing Fe(II) and Fe(III) co-localization in neuromelanin neurons (Ide-Ektessabi and Rabionet, 2005). For all these studies either the Fe histochemistry or the Fe micro-XANES analysis, samples were chemically fixed and analyzed at room temperature. In two other XANES studies where tissues were cryogenically processed, without chemical fixation, there were no significant differences in Fe oxidation state between PD patients and controls, and only Fe(III) was observed (Griffiths et al., 1999; Chwiej et al., 2007). In a study of cryogenically processed tissues from the SN of PD and control subjects, measurements using Mössbauer spectroscopy evidenced the presence of Fe in a ferritin-like form and did not detect any divalent Fe (Wypijewska et al., 2010). Our data obtained in cryogenic native conditions indicate that Fe is present only as Fe(III) in the 6-OHDA model and XANES spectra from all samples are very similar to ferritin spectrum. This result is in good agreement with XAS and Mössbauer spectroscopy data obtained on human PD cryogenically processed tissues (Griffiths et al., 1999; Chwiej et al., 2007; Wypijewska et al., 2010). Overall these results suggest that low temperature sample environment is highly advocated for micro-XAS speciation of Fe, as also suggested by previous studies of biological samples due to their sensitivity to X-ray irradiation (Bacquart et al., 2007; Ortega, 2011; Porcaro et al., 2018). Our data also indicate that Fe was present exclusively at Fe(III) oxidation state in all samples, and with a XANES spectral signature similar to that of ferritin. However, the limit of speciation of micro-XANES is in the 10 μg.g^–1^ range (Bacquart et al., 2007) meaning that minor changes in Fe oxidation state or in Fe(III) speciation, i.e., Fe(III) binding to other molecules than ferritin, would be difficult to evidence with this technique. Other analytical approaches such as electrothermal atomic absorption spectrometry have shown that Fe(III) could be present within the SN of PD patients in a more labile form than bound to ferritin, with a concentration of labile Fe in the range from 40 to 100 ng.g^–1^ (Wypijewska et al., 2010).

Another important result of this study is the higher content of Mn in the SNpc, the region rich in dopaminergic neurons, compared to the SNpr. There are some indications in the literature for the potential preferential localization of Mn in the SNpc vs. the SNpr. A SXRF image of the brain from a rat treated by intraperitoneal injections of Mn indicated that Mn content could be higher in the SNpc than in the SNpr (Robison et al., 2012). This result is very similar to our observations of rat brains, but in our case the animals were not treated with Mn. In another study of the same authors, the dopaminergic neurons of the SNpc were identified as potential target of Mn accumulation after rat exposure to excess Mn (Robison et al., 2015). Mn was clearly detected in the perinuclear region of dopaminergic neurons identified using a retrograde fluorescent tracer. This subcellular distribution is in agreement with data from our group showing the peri-nuclear accumulation of Mn in the Golgi apparatus of dopamine neurons (Carmona et al., 2010, 2014). Important questions remain open about why and by which mechanism dopaminergic neurons would accumulate preferentially Mn. A higher uptake through the increased expression of the transferrin receptor (Robison et al., 2012) or through a decreased efflux related to the expression of Mn detoxification proteins such as SLC30A10 (Carmona et al., 2019) are potential mechanisms of Mn accumulation that still need to be investigated.

In conclusion, metal dyshomeostasis in the nervous system is observed in many neurodegenerative diseases, however, the role of metal modifications in the etiological processes is still not well understood. Micro-analytical approaches are being developed to investigate the dysregulation of biological metals in well-identified regions of the brain. Thanks to the methodology presented in this article, we could investigate the distribution of biological metals in precisely identified brain regions by comparing immuno-histochemical staining and chemical imaging results showing the opposite enrichment of Fe and Mn in the SNpr and SNpc, respectively. There are still exciting experiments to perform to fully identify the sites of Mn and Fe localization in the SNpc and SNpr in normal and pathological conditions. Our data also suggest that the 6-OHDA lesioned rat model, although very helpful to study advanced stages of PD progression, is limited to investigate intermediate development stages of the disease. The increase of Fe content in the SN following 6-OHDA administration as reported in the literature is observed when almost all the dopaminergic neurons are lost. Therefore Fe increase in the SN might not be involved in the neurodegenerative process induced by 6-OHDA since we did not observe such Fe dyshomeostasis for intermediate stages of injury (35–85% of dopaminergic neurons loss). Finally, our study demonstrates the feasibility and details the methodology that could now be applied to investigate other neurodegenerative diseases when metal dyshomeostasis is suspected to be involved.

## Data Availability Statement

The datasets generated for this study are available on request to the corresponding authors.

## Ethics Statement

The animal study was reviewed and approved by Ethics committee of the Grenoble Institute of Neuroscience.

## Author Contributions

AC, MS, and RO contributed to the conception and design of the study. LP and CC prepared samples. CC performed cryo-sections and immunostaining. AC, SR, LP, and RO performed PIXE experiments. AC performed PIXE data treatment. LP, DV, and RO performed synchrotron experiments. DV and RO performed synchrotron data treatment. AC and SR performed the statistical analysis. AC and RO wrote the first draft of the manuscript. All authors contributed to the manuscript revision, and read and approved the submitted version.

## Conflict of Interest

The authors declare that the research was conducted in the absence of any commercial or financial relationships that could be construed as a potential conflict of interest.
